# New Insights into Various Production Characteristics of *Streptococcus thermophilus* Strains

**DOI:** 10.3390/ijms17101701

**Published:** 2016-10-12

**Authors:** Yanhua Cui, Tingting Xu, Xiaojun Qu, Tong Hu, Xu Jiang, Chunyu Zhao

**Affiliations:** 1 Department of Food Science and Engineering, School of Chemistry and Chemical Engineering, Harbin Institute of Technology, Harbin 150090, China; xutingting@gmail.com (T.X.); tonghu1992715@gmail.com (T.H.); jiangxu2016@gmail.com (X.J.); zhaochunyuhit@yeah.net (C.Z.); 2 Institute of Microbiology, Heilongjiang Academy of Sciences, Harbin 150010, China; qvxiaojun@163.com

**Keywords:** *Streptococcus thermophilus*, genomics, plasmid, production characteristics

## Abstract

*Streptococcus thermophilus* is one of the most valuable homo-fermentative lactic acid bacteria, which, for a long time, has been widely used as a starter for the production of fermented dairy products. The key production characteristics of *S. thermophilus*, for example the production of extracellular polysaccharide, proteolytic enzymes and flavor substances as well as acidifying capacity etc., have an important effect on the quality of dairy products. The acidification capacity of the strains determines the manufacturing time and quality of dairy products. It depends on the sugar utilization ability of strains. The production of extracellular polysaccharide is beneficial for improving the texture of dairy products. Flavor substances increase the acceptability of dairy products. The proteolytic activity of the strain influences not only the absorption of the nitrogen source, but also the formation of flavor substances. Different strains have obvious differences in production characteristics via long-time evolution and adaptation to environment. Gaining new strains with novel and desirable characteristics is an important long-term goal for researchers and the fermenting industry. The understanding of the potential molecular mechanisms behind important characteristics of different strains will promote the screening and breeding of excellent strains. In this paper, key technological and functional properties of different *S. thermophilus* strains are discussed, including sugar metabolism, proteolytic system and amino acid metabolism, and polysaccharide and flavor substance biosynthesis. At the same time, diversity of genomes and plasmids of *S. thermophilus* are presented. Advances in research on key production characteristics and molecular levels of *S. thermophilus* will increase understanding of molecular mechanisms of different strains with different important characteristics, and improve the industrialization control level for fermented foods.

## 1. Introduction

*Streptococcus thermophilus* is the only streptococcal species widely used in food fermentations, especially for yogurt manufacturing. It has been used as a seed along with *Lactobacillus delbrueckii* subsp. *bulgaricus* to produce yogurt for thousands of years. At the same time, *S. thermophilus* has also been used in various artisanal and industrial dairy products, for example some cheeses and fermented milk [1,2]. It can improve the texture and flavor properties of these dairy products, and accelerate the acidifying rate of dairy products. Additionally, *S. thermophilus* is found to have various probiotic effects, including antioxidant activities, modulation of intestinal microbiota, inhibition of specific pathogens etc. [1,2]. This species has attracted broad interest in past decades because of its industrial application and probiotic effects.

The production characteristics of *S. thermophilus*, such as acidifying capacity, proteolytic activity, fast growth, production of exopolysaccharide (EPS), bacteriocins and flavor substances, antiphage, and host defense ability etc. directly or indirectly affect the quality of fermented dairy products [2,3,4,5,6,7]. The acidifying capacity, proteolytic activity, EPS and flavor production abilities are key production characteristics of strains. The rate of acidification is the most important criteria for evaluating whether or not *S. thermophilus* is a good starter culture for use in fermented dairy products. It determines the fermenting time and quality of fermented dairy products [2,4,6]. The acidifying capacity of strains is closely related with their sugar utilization ability. EPSs play an important role in improving the viscosity, texture and mouthfeel of dairy products [2,3,4,7]. Therefore, high EPS production is one of the most important and attractive properties of *S. thermophilus* strains. Flavor is a key factor determining the acceptability and preference of dairy products. The proteolytic system of *S. thermophilus* can degrade casein, which is an important precursor of flavor compounds. Furthermore, the proteolytic activity of strain has a tight correlation with its acidifying capacity [6].

In order to adapt to different environments, genomes of different *S. thermophilus* strains were found to change during evolution, and form unique genetic compositions and special corresponding control systems. For example, some excellent industrial strains genomes contain distinctive *eps* gene clusters that are responsible for EPS production [2,3,4,7]. Strains also show different fermentation characteristics, including the ability to produce acid, adaptability to environmental stress, protein decomposition ability, sugar utilization ability, EPS and bacteriocin production abilities etc. [3,4,5,6,7,8,9].

At present, most studies on *S. thermophilus* focus on the analysis of diversity based on phenotypic characteristics [3,4,5,6,7,8,9]. However, emerging tools for synthetic biology have led to a number of strategies for metabolic engineering. The potential molecular mechanisms behind important characteristics of different strains have been partially reported.

## 2. Diversity of Genomes and Plasmids of *S. thermophilus*

### 2.1. Diversity of S. thermophilus Genomes

The genome sequence information of some strains of *S. thermophilus* was recently published [10,11,12,13,14,15,16,17,18]. This accelerates research on *S. thermophilus* at the molecular level. The entire genomes of 14 *S. thermophilus* strains (LMG18311, JIM8232, CNRZ1066, LMD-9, MN-ZLW-002, ND03, ASCC 1275, SMQ-301, MN-BM-A02, MN-BM-A01, S9, KLDS MS, CS8, and KLDS 3.1003) have been fully sequenced [10,11,12,13,14,15,16,17,18]. These strains were all isolated from fermented milk or milk [10,11,12,13,14,15,16,17,18]. *S.* *thermophilus* strains MTH17CL396, M17PTZA496, TH1435, TH1436, TH982, TH1477, 1F8CT, and TH985 have been completely sequenced at the chromosome level [19,20,21]. Meanwhile, some genome strains have finished draft, including CNCM I-1630, MTCC5460, MTCC5461, DGCC7710, and C106 [22,23,24]. The genomes range in size from 1.609–2.065 Mb with 1476–2193 proteins.

The evolution of *S.* *thermophilus* in milk environment is mainly via gene horizontal transfer and natural competition. In the process of evolution, some carbohydrate metabolism genes, virulence genes and other genes gradually degrade and disappear in the stable milk environment [6,25,26,27]. The virulence genes in other streptococci are either non-functional or completely absent in *S. thermophilus* [6,25,26,27]. *S. thermophilus* contains genes for glycolytic pathway, the non-oxidative branch of the pentose phosphate pathway, and pyruvate metabolism. It does not have the pyruvate carboxylase-encoding gene, which is required for the formation of oxaloacetate from pyruvate. Functional gene distribution analysis of the open reading frame group showed that the *S.* *thermophilus* LMG18311, LMD-9 and CNRZ1066 strains form obvious functional clusters, which specifically adapt to the nutrition of the milk environment [25]. *S.* *thermophilus* strains show obvious differences to other *Streptococcus* species in genome [6,25,26,27].

Some research indicates that the proteolytic system, nitrogen metabolism, sugar utilization and transporter systems of *S. thermophilus* are very important during the adaptation to milk environments [3,6,15,25,28]. Research on 47 strains of *S. thermophilus* isolated from dairy by means of comparative genome hybridization analysis has found that the core genes of the different strains genomes are different. The differences of core genes were mainly the biosynthesis of bacteriocin and EPS, peptide metabolism, and phage resistance related genes etc. [28]. This evolution has led to the various metabolic activities of different *S.* *thermophilus* strains [3,4,5,6,7,8,9].

### 2.2. Plasmid Diversity of S. thermophilus

As a self-replication DNA molecule, plasmids do not have the necessary genetic material for the survival of bacteria, but they often carry some special genes which confer important traits for strains, for example, casein utilization, EPS biosynthesis, potassium transport, bacteriophage resistance, and bacteriocin production related genes etc. [29]. Compared with some lactic acid bacteria (LAB) that contain plenty of plasmids, for example *Lactobacillus plantarum* and *Lactococcus* *lactis* etc., *S. thermophilus* strains carry very few plasmids (Table A1, http://www.ncbi.nlm.nih.gov) [29,30].

Most *S. thermophilus* strains do not contain plasmids. Some strains have no more than 2 plasmids, which range in size from 2.67 to 8.14 kb. To date, most plasmids of *S. thermophilus* have no apparent phenotypic traits. Some plasmids encode small heat shock proteins, including pER341 [31], pCI65st [32], pND103 [33], pST04 and pER1-1 [34], pt38 [35], pER7, pER16, pER26, pER35, pER36, and pER41 [36,37]; p2, pK1002C2, and pK2007C6 [12]. Amino acid sequence comparisons of the small heat shock proteins and of the replication proteins from pER16, pER35, and pER36 revealed a high degree of identity suggesting a common origin [37]. Some research indicates that small heat shock proteins are induced by elevated temperatures and low pH, and expression of these proteins can increase the viability of bacteria in extreme environments [34,37]. Therefore the promoter of heat shock protein gene *hsp16.4* of pER341 is under investigation for potential use in temperature-controlled expression of heterologous genes in LAB [31]. There are restriction modification system-related genes in plasmids pCI65st [32], pSt08, pSt0 [34], and pER35 [37].

Generally plasmids replicate via the rolling circle replication (RCR) and θ mode of replication. The mode of replication of plasmids has a close relationship with some important characteristics of plasmid-derived vectors, including host range, stability, and copy number etc. [29]. RCR plasmids are usually small in size (less than 10 kb), have multiple copies, and are tightly organized. However, θ plasmids have better structural stability than RCR plasmids. Most plasmids from *S.* *thermophilus* replicate via RCR. Most RCR plasmids of *S.* *thermophilus* belong to the pC194 family, however pSMQ172 has been assigned to the pE194/pMV158 family [38]. Although plasmids pSMQ-316 and pSMQ-312b are less than 10 kb, they replicate via θ-replicating mode [39].

## 3. Technological and Functional Properties of *S. thermophilus*

### 3.1. Sugar Metabolism

**Figure 1:**
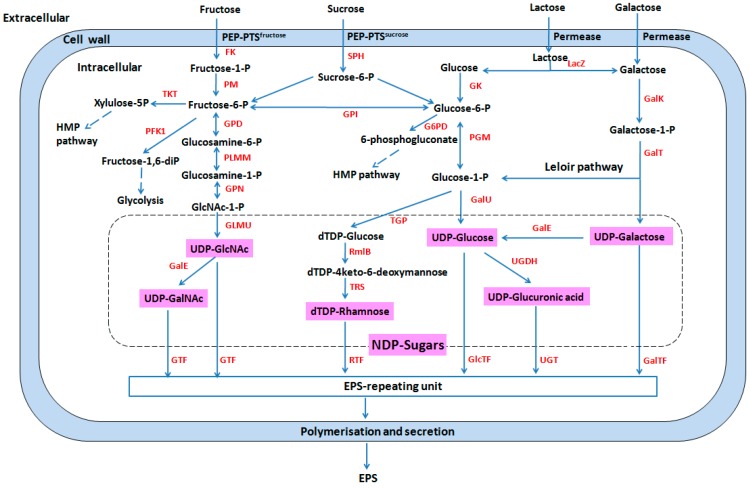
Pathways of sugar metabolism and exopolysaccharide (EPS) biosynthesis in *S. thermophilus*. FK: fructokinase; G6PD: glucose-6-phosphate dehydrogenase; GalE: UDP-galactose-4-epimerase; UGDH: UDP-glucose 6-dehydrogenase; GalK: galactokinase; GalT: galactose-1-phosphate uridylyltransferase; GalTF: galactosyltransferase; GalU: UDP-glucose pyrophosphorylase; GK: glucokinase; GlcTF: glucosyltransferase; GLMU: *N*-acetylglucosamine-1-phosphate uridyltransferase; GPD: glucosamine-6-phosphate deaminase; GPI: glucose-6-phosphate isomerase; GPN: glucosamine-1-phosphate *N*-acetyltransferase; GTF: glycosyltransferase; HMP pathway: pentose phosphate pathway; LacZ: β-glalactosidase; MPI: mannose-6 phosphate isomerase; NDP sugars, nucleotide sugars; PEP: phosphoenolpyruvate; PFK1: phosphofructokinase; PGM: α-phosphoglucomutase; PLMM: phosphoglucosamine mutase; PM: phosphomutase; PTS: sugar phosphotransferase system; RmlB: dTDP-glucose 4, 6-dehydratase; RTF: rhamnosyltransferase; SPH: sucrose-6-phosphate hydrolase; TGP: dTDP-glucose pyrophosphorylase; TKT: transketolase; TRS: dTDP-4-dehydrorhamnose 3,5-epimerase; UDP-GalNAc: UDP-*N*-acetylgalactosamine; UDP-GlcNAc: UDP-*N*-acetylglucosamine; UGT: UDP-glucuronosyltransferase. Double headed arrow indicates the bidirectional reaction; dashed arrows indicate a part pathway; arrow indicates a reaction; red fonts indicates enzyme; words with red background color indicate the NDP sugars.

In industrial fermentation processes, the rates of milk acidification by *S.* *thermophilus* species are of major technological importance. The acidification rate of strains depends on their sugar utilization ability. A number of works indicate that utilization of glucose, lactose and fructose by *S. thermophilus* is a common characteristic in all existing studies, while galactose, mannose, sucrose, maltose, melibiose, and raffinose utilizations have variable profiles [40,41,42,43,44,45,46,47]. At the same time, it has been found that carbohydrate metabolism is essential for the colonization of *S. thermophilus* in the digestive tract of gnotobiotic rats [48]. Colonization ability is an important determining factor for developing bacterial health function in humans. A detailed description of sugar metabolism and central carbon pathways in *S. thermophilus* is presented in Figure 1.

*S. thermophilus* prefers lactose over glucose as a major carbon and energy source. The *lac* operon controls the transport and hydrolysis of lactose, and its transcription is induced during growth on lactose. It encodes lactose permease (LacS) and cytoplasmic β-galactosidase (LacZ). Lactose is cleaved into glucose and galactose by LacZ. Glucose is phosphorylated by glucokinase to glucose-6-phosphate, and then is further utilized through the glycolysis pathway. Galactose is converted to glucose-1-phosphate by means of the Leloir pathway, which consists of the regulator GalR, galactokinase (GalK), galactose-1-phosphate uridyltransferase (GalT), UDP-glucose-4-epimerase (GalE), and galactosemutarotase (GalM). They were found to be organized in the *galRKTEM* gene cluster [49,50].

The *gal* promoter plays an important role in the galactose phenotype of *S. thermophilus* [42,50,51,52,53,54]. It has been found that the point mutations in the promoter region of *gal* operon lead to low expression level of the Leloir pathway enzymes for galactose utilization [42,50]. At the same time, some studies demonstrate that the low level of galactokinase (GalK) activity in galactose negative strains with respect to galactose positive strains indicate poor *galK* translation, which was related to nucleotide differences in the ribosome binding site [42,51]. One class of Gal-positive mutants described by Vaughan et al. contained a G-to-A substitution in the −10 box of *galK* that turned out to be a promoter up mutation [50]. Recently, it was found that spontaneous galactose-fermenting mutant St1-Ga^+^-1 has the same mutation in the −10 region of *galK*, i.e., TACAAT, which is closer to the consensus sequence TATAAT. Strain St1-Ga^+^-1 can ferment galactose, while its mother strain St1-WT is unable to utilize galactose, although it has intact *galKTEM* genes [54]. At the same time, Sørensen et al. found that another spontaneous galactose-fermenting mutant St2-Ga^+^-1 had a C-to-A mutation between the −10 region and the *galK* transcription initiation site [54]. It was speculated that this mutation might increase the “opening potential” for the −9 to +3 region, and then lead to creating a stronger promoter [54].

The utilization of galactose is a desirable property for strains used in industrial dairy fermentations. Galactose accumulation leads to the growth of undesirable LAB in milk products, and cheese browning during baking. Gal-positive *S. thermophilus* strains can inhibit the growth of undesirable LAB, and prevent the browning defects [40,41,42,43]. Furthermore, galactose utilization enhances EPS production. Galactose utilization can produce nucleotide sugars, which are EPS precursors and whose low level might be a potential bottleneck in EPS production [55,56]. At the same time, high level of galactose consumption may lead to accumulation of toxic galactitol in human tissue cells [57]. Galactose utilization in strains decreases galactose content in dairy products, which is beneficial for human health.

### 3.2. Polysaccharide Biosynthesis

Extracellular polysaccharides are produced by a variety of bacteria, and are present as capsular polysaccharides (CPSs) or EPSs. The former are tightly linked to the surface of the microbial cell, the latter are released into the growth medium during bacteria growth and are not attached permanently to the cell surface. Most *S.* *thermophilus* strains can synthesize EPSs [58]. Additionally some *S. thermophilus* strains may produce CPSs [59].

EPS production is one of the most important properties of *S. thermophilus*, especially dairy strains. EPSs act as in situ-produced natural biothickeners that can improve the viscosity, texture and mouthfeel of the dairy products, and prevent syneresis in yogurt [60,61,62]. EPSs from *S. thermophilus* benefit the health of host animals, and enhance the immune responses of host animals [63,64,65,66]. It is speculated that immunoregulatory effects of EPSs are related to their chemical composition.

EPSs are long-chain polysaccharides consisting of branched, repeating units of sugars or sugar derivatives. They are classed into homopolysaccharides that consist of a single type of sugar, and heteropolysaccharides with repeating units consisting of different sugars. Most *S. thermophilus* strains synthesize heteropolysaccharide [58]. *S. thermophilus* EPSs are predominantly composed of galactose, glucose, and rhamnose in different ratios. Additionally, acetyl-galactosamine, fucose, and acetylated galactose moieties have also been found in *S. thermophilus* EPSs [67,68]. Generally, the structures of EPS have a close relationship with their functions. Therefore, the highly polymorphic structures of EPSs maybe confer a broad application potential [3,69]. A detailed description of EPS biosynthesis in *S. thermophilus* has been presented in Figure 1.

Sugar nucleotides act as precursor molecules of EPSs, and offer sugar residues in the glycosylation reactions that produce polysaccharides [70,71]. The biosynthesis of EPSs is regulated and determined by *eps* gene cluster. EPS production is associated with plasmids in *L. lactis* [72]. However, all *eps* gene clusters of *S.* *thermophilus* are located on the chromosome. Generally, the *eps* gene clusters contain genes involved in regulation of EPS production (*epsA*, *epsB*), chain-length determination of EPS (*epsC*, *epsD*), formation of the repeating units *(epsE*, *epsF*, *epsG*, *epsH*, and *epsI*), and EPS polymerization and export (*epsK*, *epsL*, and *epsM*) [58,73]. The 5’ end of *eps* gene clusters is the *deoD* gene, which putatively encodes purine nucleotide phosphorylase and is presumably involved in the biosynthesis and catabolism of nucleotides. It has been documented that the *orf14.9* gene distributed downstream of most *eps* gene clusters is associated with the cell growth of *S.* *thermophilus* [7].

The structural diversity of EPSs molecules is correlated with the genetic diversity of the *eps* locus. At least 20 distinct types of *eps* gene clusters were found in 51 *S. thermophilus* strains known to date [7,15,16,17,18,19,20,21,22,23,24]. The diversity of *eps* gene cluster may have a direct effect on the EPS production capacity of the strain. To date, distribution of regulatory and structural genes is conserved in known *eps* cluster of *S. thermophilus* strains [7,15,70].

Sugar nucleotides are assembled into a repeating unit by means of multiple glycosyltransferases (GTFs). At least 67 GTFs have been found in 51 *S. thermophilus* strains known to date [7,15,16,17,18,19,20,21,22,23,24]. GTF determines the monosaccharide composition of EPS. Indeed, the number of GTFs present in *eps* gene clusters will correspond more or less to the number of monosaccharide units present in the various structures of the corresponding heteropolysaccharides repeating units [74,75,76].

Recently a novel *eps* cluster *deoDepsAB1C1DEFGHIJ2C2DKLMNOrf14.9epsOPQ* was identified in *S. thermophilus* ASCC 1275 (ST 1275) [15]. The strain produces about 1029 mg/L in milk medium in the presence of 0.5% whey protein concentrate [77]. This is the highest known EPS yield of all the reported data on *S. thermophilus*. The ASCC 1275 strain produced not only CPS but also EPS. The former does not cause ropiness in milk products, whereas the latter contributes to the enhanced texture of milk products [62]. The EPS produced from ST 1275 exhibited texture modifying properties in Mozzarella cheese and yogurt [62,78,79].

The *eps* gene cluster of ASCC 1275 contains highly conserved regulatory genes (*epsA*, *epsB*) and chain-length determination related genes (*eps1C*, *eps1D*) [15]. At the same time, this cluster has GTF genes (*epsE*, *epsF*, *epsG*, *epsH*, *epsI*, *epsJ* and *epsK*), which may transfer various nucleotide sugars including UDP-glucose, UDP-galactose, dTDP-rhamnose, UDP-GlcNAc and UDP-galactofuranose to form the repeating units [15]. Interestingly, an additional *eps2C–eps2D* was found in this cluster, which was probably involved in the chain length determination. Additionally, the *eps* cluster contains a unique UDP-galactopyranose mutase, which is responsible for the synthesis of UDP-galactofuranose [15]. It is rarely found in the *S. thermophilus* *eps* gene cluster [18].

The EPS production ability of *S. thermophilus* is affected by two factors, i.e., strain dependent and cultivation conditions, such as temperature, carbon sources, nitrogen sources, and pH, etc. [77,80,81]. In general, the amount of EPS produced by *S. thermophilus* is at relatively low levels, i.e., 50 to 400 mg/L in milk medium.

The EPS yield of *S. thermophilus* 05-34 reaches 250 mg/L in 10% reconstituted skim milk with 80 g/L sucrose and 30 g/L soy peptone at an initial pH 7.0 and 37 °C for 30 h [81]. The research indicated that the molecular mass of EPS is 4.7 × 10^5^ Da, which is increased by 9 times compared with non-optimal fermentation conditions, while monosaccharide composition does not change. Additionally, real-time quantitative PCR analysis indicated that the transcription level of chain length determination related gene *epsC* was up-regulated 2.7-fold. It was speculated that the improved degree of polymerization of monosaccharide led to increasing molecular mass of EPS. The results demonstrated that the optimized fermentation conditions can not only increase the EPS yield, but also improve EPS molecular mass.

Instability of EPS production and variability of polymer yields are common in the dairy industry. Generally, it is thought that this genetic instability is due to loss of the encoding *eps* genes plasmid in *L. lactis*. However, it is speculated that insertion sequences (IS) or transposase genes lead to the instability in *S. thermophilus* [73]. The *eps* gene cluster of some strains contains transposable elements, such as IS sequences or transposase genes. *S. thermophilus* MN-ZLW-002 was isolated from a traditional fermented dairy food, and has a prominent EPSs production ability [14]. Interestingly, its *eps* gene cluster contains 9 transposases genes. The *eps* cluster of *S. thermophilus* CNRZ368 consists of 32.5 kb, contains 25 ORF (open reading frame) and six intact or truncated ISs belonging to four different families, ISS1, IS981, IS1193 and IS1194 [73]. *S. thermophilus* ND03 genome carries a unique 23.4-kb *eps* gene cluster (STND_1010 to STND_1035), which contains 10 EPS-related genes (*epsA*, *epsB*, *epsC*, *epsD*, *epsE*, *epsF*, *epsG*, *epsI*, *epsJ*, and *epsP*) and seven intact or truncated ISs [13]. The *eps* clusters of *S. thermophilus* CNRZ1066 and LMG 18311 have 2 and 3 transposase genes, respectively [10]. These ISs or transposases increase the instability of EPS production by means of the transposition of mobile elements. Additionally, instability of EPS production in *S. thermophilus* is related to generalized genomic instability [82].

### 3.3. Proteolytic System and Amino Acid Metabolism

The growth of LAB is limited due to the low free amino acids in milk. Milk contains an abundance of proteins. Therefore, the strains must utilize proteins, produce peptides and amino acids to satisfy its nitrogen source requirement during rapid growth in milk. The protein hydrolysis system provides the basic amino acids for the growth of the cells, and also affects the sensory characteristics and flavor of fermented dairy products [83].

The proteolytic system of LAB mainly consists of (i) an extracellular cell anchored protease capable of casein hydrolysis; (ii) a set of amino acid and peptide transport systems required for import of amino acids and (iii) a set of intracellular peptidases involved in the hydrolysis of casein-derived peptides essential for various housekeeping processes peptides transporters, and intracellular peptidases [2,4,25,84]. Recently, analysis of LAB genome sequences revealed the presence of up to 40 putative proteolytic enzymes per bacterial species, the role of more than half of them being still unknown [15,16,17,18,19,20,21,22,23,24,83]. In addition, recent data indicate that proteolysis at LAB surface is much more intense than expected so far.

**Figure 2:**
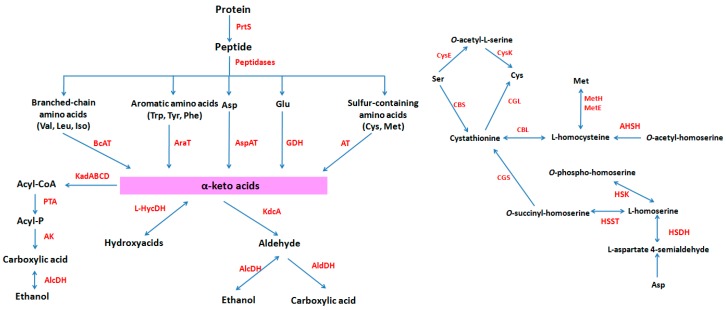
Proteolytic system and amino acid metabolism in *S. thermophilus*. AHSH: *O*-acetylhomoserine sulfhydrylase; AK: acyl kinase; AlcDH: alcohol dehydrogenase; AldDH: acetaldehyde dehydrogenase; AraT: aromatic aminotransferase; Asp: aspartate; AspAT: aspartate transaminase; AT: aminotransferase; BcAT: branched-chain aminotransferase; CBL: cystathionine γ lysase; CBS: cystathionine β synthase; CGL: cystathionine β lysase; CGS: cystathionine γ synthase; Cys: cysteine; CysE: serine *O*-acetyltransferase; CysK: *O*-acetylserine sulfhydrylase; GDH: glutamate dehydrogenase; Glu: glutamate; HSDH: homoserine dehydrogenase; HSK: homoserine kinase; HSST: homoserine *O*-succinyltransferase; Iso: isoleucine; KadABCD: keto acid dehydrogenase complex; KdcA: keto acid decarboxylase; Leu: leucine; l-HycDH: l-hydroxyacid dehydrogenase; Met: methionine; MetE: homocysteine methyltransferase; MetH: homocysteine *S*-methyltransferase; Phe: phenylalanine; PTA: phosophotransacylase; Ser: serine; Trp: tryptophan; Tyr: tyrosine; Val: valine. Double headed arrow indicates the bidirectional reaction; arrow indicates a reaction; red fonts indicates enzyme; words with red background color indicate the important intermediate products (α-keto acids).

The proteinase type and proteolytic activity of LAB have obvious differences [85,86]. At present, it is found that five types of extracellular proteinases in LAB, including PrtP (*L. lactis*, *Lactobacillus paracasei*), PrtH (*Lactobacillus helveticus*), PrtR (*Lactobacillus rhamnosus*), PrtS (*S. thermophilus*), PrtB (*L. delbrueckii* ssp. *bulgaricus*) [83]. The proteolytic system of *S. thermophilus* comprises more than 20 proteolytic enzymes, including cell-wall bound proteinase PrtS, endopeptidases (PepO, PepF), dipeptidases (PepD, PepV), tripeptidase PeoT, and proline peptidases (PeoX, PeoP, PepQ) etc. (Figure 2).

Additionally, there are great differences in the proteolytic activity of the different strains [6,47,87,88,89]. Galla et al. (2009) evaluated proteolytic and acidifying properties of 30 *S. thermophilus* strains isolated from yoghurt or cheeses [6]. Among 30 strains tested, 12 exhibited cell envelope-associated proteinase activity (PrtS^+^), three displayed slight PrtS activity (PrtS^+/−^). Interestingly, 8 strains had no proteinase activity, though they contained corresponding gene *prtS*. It is speculated that the absence of PrtS activity in the PrtS^−^ strain probably results from an alteration of the *prtS* regulation.

The previous studies indicated that there was a tight correlation between the high acidifying capacity and high proteolytic activity of strains [6,89]. These results indicated that the presence of high proteinase activities allowed strains to grow and produce acid more rapidly in milk. The types of 16S–23S intergenic spacer (ITS) region sequences were related with the same phenotypic properties [6]. At present, four types were found in *S. thermophilus*, including ITS-St-I, ITS-St-II, ITS-St-III, and ITS-St-IV [90]. ITS-St-I and ITS-St-II types are common types. Galia et al. found that most strains had an ITS-St-I allele (17 out of 30) or an ITS-St-II allele (10 out of 30) while only three exhibited an ITS-St-V allele [6]. Generally, strains with an ITS-St-II allele exhibited the high proteolytic and acidifying capacities, whereas strains with an ITS-St-I allele displayed slight PrtS activity or no PrtS activity [6].

### 3.4. Flavor

Flavor is one of the most important properties of food products, and is a key factor in determining acceptability and preference [91]. More than 100 different flavor compounds are found in yogurt, including alcohols, aldehydes, ketones, acids, esters, lactones, sulfur-containing compounds, and heterocyclic compounds etc. [91,92]. These flavor compounds include the volatiles already present in the milk and specific flavor compounds produced from milk fermentation [92,93,94]. Lactic acid, acetaldehyde, diacetyl, acetoin, acetone, and 2-butanone are important flavor compounds, and are responsible for typical aroma and flavor of yogurt [91,92,93,94].

LAB play an important role in flavor formation during the fermentation of dairy products. Flavor-forming capacity is an important evaluating indicator of starter culture in fermented dairy. The flavor of dairy products originated from protein, fat and carbohydrate in the milk. Casein is the main precursor of flavor compounds when LAB are growing in milk [94]. Casein is degraded into its constituent amino acids by means of proteolytic system of LAB. Then, free amino acids are converted to various flavor compounds, such as aldehydes, alcohols, and esters [91,94]. The flavor compounds are mainly from the branched-chain amino acids, the aromatic amino acids, and the sulfur-containing amino acids [94,95,96,97]. These amino acids convert into flavor compounds mainly via transamination route, which uses an α-keto acid as an amino group acceptor for the amino transferases. Some enzymes involved in the transamination route of amino acid degradation have been found in *S. thermophilus* genomes, including branched-chain aminotransferase (BcAT), glutamate dehydrogenase (GDH), alcohol dehydrogenase (AlcDH), keto acid dehydrogenase complex, phosphotransacylase (PTA), l-hydroxyacid dehydrogenase (l-HycDH), and esterase A (EstA) [10,12,94]. Research indicates that *S. thermophilus* exhibits glutamate dehydrogenase activity, which produces α-ketoglutarate from glutamate, and consequently is capable of catabolizing amino acids in the reaction medium without α-ketoglutarate addition [98].

The level of flavor compounds could be improved using molecular biotechnology. The ability to generate important metabolites of *S. thermophilus* is improved by expression of α-acetolactate synthase gene (*als*) and alcohol dehydrogenase gene (*adhB*) [99]. High levels of 2,3-butanediol and ethanol are obtained by over expressing the *als* gene or *adhB* gene in *S. thermophilus* [99]. These metabolites improve the flavor of dairy product.

Extraction and analysis of flavor compounds via traditional methods is time-consuming. Recently fermentation time, acid and flavor substance formation of 43 strains of *S. thermophilus* were evaluated. At the same time, a multilocus sequence typing analysis of 8 functional genes associated with production of acetaldehyde and diacetyl was performed including serine hydroxymethyltransferase gene, acetate kinase gene, l-lactate dehydrogenase gene, pyruvate decarboxylase gene, serine hydroxymethyltransferase gene, NADH oxidase gene, pyruvate formate-lyase gene, and pyruvate dehydrogenase gene. It was found that the grouping based on functional genotype were consistent with that of derived from the phenotyping characteristics [100]. A similar result was obtained in the research on *L. delbrueckii* ssp. *bulgaricus* [101]. The results indicated that functional gene multilocus sequence typing technology can be used to predict the fermentation and flavor-producing characteristics of yogurt-producing bacteria [100,101].

## 4. Conclusions

Sugar utilization ability, EPS and flavor substance production ability, and proteolytic activity are important production characteristics of *S. thermophilus*. They determine the quality of fermented dairy products. Different *S. thermophilus* strains show diversity in their production characteristics. A great deal of information on genomes and plasmids of *S. thermophilus* has been published. This accelerates people’s understanding of the potential molecular mechanisms behind important characteristics of different strains. Generally, the different strains with different production characteristics have obvious differences in their genomes. The differences are mainly involved in the biosynthesis of bacteriocin and EPS, peptide metabolism, and phage resistance related genes etc.

The sugar utilization ability of strains determines the acidification rate of fermented milk. The *gal* promoter plays an important role in the galactose utilization of *S. thermophilus*, which is a desirable property for strains. The point mutations in the promoter region of *gal* operon lead to low expression level of galactose utilization related enzymes. EPS can improve the texture of fermented milk products. The *eps* gene cluster is responsible for the biosynthesis and regulation of EPS. The *eps* gene clusters of *S. thermophilus* have abundant genetic diversity, which has a close relationship with the structure and yield of EPSs. PrtS is most important proteinase of *S. thermophilus*. Most strains contain *prtS* gene. However, some *prtS* genes do not have activity because of the alteration of the *prtS* regulation. Casein is the main precursor of flavor compounds in fermented milk products. The proteolytic system plays a crucial role in the formation of flavor compounds. Although the potential molecular mechanisms behind important characteristics of different strains have been reported partially, further research is needed in order to reveal the full molecular mechanisms.

## Abbreviations

AlcDH	Alcohol dehydrogenase
BcAT	Branched-chain aminotransferase
CPSs	Capsular polysaccharides
EPSs	Exopolysaccharides
EstA	Esterase A
GDH	Glutamate dehydrogenase
GTFs	Glycosyltransferases
IS	Insertion sequence
LAB	Lactic acid bacteria
l-HycDH	l-hydroxyacid dehydrogenase
PTA	Phosphotransacylase
RCR	Rolling circle replication

## Appendix A

**Table A1:** The plasmids of *Streptococcus thermophilus*.

Plasmid Name	Strain	Replication	Size (kb)	(G+C)%	Protein	Gene	Pseudogene	Rep Protein	Mob Protein	Function	NCBI Accession	Ref.	Source
pK1002C2	K1002C2		3.38	35	2	2	-	RepA 314 aa		Small heat shock protein	NC_019231.1	[12]	
pK2007C6	K2007C6		2.98	35.1	2	2	-	RepA 314 aa		Small heat shock protein	NC_019232.1	[12]	
pSTER_A	LMD-9	RCR	4.45	37	4	4	-			DNA segregation ATPase FtsK/SpoIIIE or related protein	NC_008500.1	[25]	Yogurt
pSTER_B	LMD-9	RCR	3.36	35.1	2	2	-	Rep 314 aa		Small heat shock protein	NC_008501.1	[25]	Yogurt
pER341	ST134	RCR	2.798	33.7		2					AF019139.1	[31]	An in-house culture collection
pCI65st	NDI-6	RCR	6.5	34.5	5	5		RepA 315 aa		Small heat shock protein; enolase	AF027167.1	[32]	Indian fermented milk dahi
pND103	ST2-1		3.53	32.4	4	4	-				NC_004747.1	[33]	
pSt0	St0	RCR	8.1	37	6	6				Cytosine-specific methyltransferase; type II restriction endonuclease	NC_025154	[34]	Dairy product
pSt04	St04	RCR	3.1							Small heat shock protein	AJ242477	[34]	Dairy product
pSt08	St08	RCR	7.51		9	1		Rep 313 aa		Putative adenine specific methyl transferase	AJ 239049	[34]	Dairy product
pSt106			5.283	36		1		Rep 287 aa		Putative resolvase	AJ 242479	[34]	Dairy product
pJ34	J34	RCR	3.38			1		RepA 315 aa			AJ242475	[34]	Dairy product
pSt22-2	St22											[34]	Dairy product
pER1-1		RCR	3.365		2	1		RepA 314 aa		Small heat shock protein	AJ 242476	[34]	Dairy product
pER1-2			4.45	36.9	5	5					NC_025196.1	[34]	Dairy product
pt38	ST2783		2.91	32.4	5	9	-	Rep 311 aa			NC_005098.1	[35]	Bulgarian yogurt
pER16			4.27		3			Rep 315 aa		Small heat shock protein	AF177166	[37]	
pER35	ST135		9.53	36.5	5	5	-	RepA 315 aa		Small heat shock protein; type IC restriction subunit; type IC modification subunit; type IC modification subunit	NC_000937.1	[37]	
pER36	ST136		3.5	34.4	2	2	-	RepA 315 aa		Small heat shock protein	NC_000938.1	[37]	
pSMQ172	SMQ-172	RCR	4.23	38	4	5	-	Rep 223 aa	Mob 499 aa		NC_004958.1	[38]	
pSMQ-316		θ	6.71	37.7	5	5	-			Primase-helicase; integrase	NC_010859.1	[39]	
pSMQ-312b		θ										[39]	
pSMQ173b	SMQ-173	RCR	4.45	37	5	5	-	Rep 146 aa			NC_005323.1	[102]	
pSMQ-308			8.14	37.8	6	6	-				NC_005322.1	[102]	
pER371	ST371		2.67	38.2	3	3	-	Rep 247 aa			NC_004968.1	[103]	
pER13	ST113	RCR	4.14	38.4	4	4	-	RepB 217 aa	Mob 499 aa		NC_002776.1	[104]	

RCR, rolling circle replication.
